# Commentary on “Beta-Lactam Antibiotics as A Possible Novel Therapy for Managing Epilepsy and Autism

**Published:** 2018

**Authors:** Liqin ZHU, Huayu WANG

**Affiliations:** 1 Tianjin First Central Hospital, Tianjin, 300192, China; 2 The Second Hospital of Tianjin Medical University, Tianjin 300211, China

**Dear** **Editor-in-Chief**

I wrote this letter for discussing with the author of the paper titled “Beta-Lactam antibiotics as a possible novel therapy for managing epilepsy and autism, a case report and review of literature” (1). This paper is very interesting because beta-lactam antibiotics were regarded as a kind of drugs, which could cause epilepsy all the time (2-4). 

The mechanism of cephalosporin-induced convulsions is as follows: The inhibition of an inhibitory response caused by beta-lactam antibiotics is because that they bind directly to the receptor and inhibit GABA-induced Cl^-^ currentsd. β-lactam antibiotics produce convulsions via their GABA A antagonist properties. Alternatively, cephalosporins might induce convulsions by enhancing glutamate-mediated excitatory neurotransmission, possibly by activating NMDA receptors, a subtype of glutamate receptors that have an important role in regulating seizure activity (5).

In that paper, the boy was taken cefixime 200 mg/day to control diarrhea about 2 yr ago. The seizure episodes were dramatically decreased 3 days after starting the medication while his anti-epileptic medication regime was continuous. “Whenever cefixime was not administered, seizure episodes happened again. Some antibiotics such as minocycline may decrease the epileptic seizure through anti-inflammatory effects” (6). Therefore, the author contribute this anti-epilepsy effect to possible explanation of cefixime.

However, besides cefixime, the reported 9-yr-old boy was taken multiple different medications such as phenobarbital, sodium vadporate, and carbamazepine with sufficient dosages and durations. “Elevated carbamazepine levels have been reported in postmarketing experience when cefixime is administered concomitantly” (7).The antibiotic cefixime, the patient used, could induce drug-drug interaction (DDI) to increase anti-epilepsy drug level. Therefore, we guess his anti-epilepsy is likely to attribute to DDI between cefixime and anti-epilepsy drugs, but not because of cefixime.

To summary, the author provided us a new thinking to see the relationship between cephosporins and epilepsy. 
